# Quality of primary health care in Poland from the perspective of the physicians providing it

**DOI:** 10.1186/s12875-016-0550-8

**Published:** 2016-11-04

**Authors:** Anna Krztoń-Królewiecka, Marek Oleszczyk, Willemijn LA Schäfer, Wienke GW Boerma, Adam Windak

**Affiliations:** 1 Department of Family Medicine, Chair of the Department of Internal Medicine and Gerontology, Jagiellonian University Medical College, 4 Bochenska Street, 31 061 Krakow, Poland; 2 NIVEL, Netherlands Institute for Health Services Research, PO Box 1568, 3500 BN Utrecht, Netherlands

**Keywords:** Primary care, General practice, Quality of care, Health policy, Health services research

## Abstract

**Background:**

Primary care (PC) allows patients to address most of their health needs and is essential for high quality healthcare systems. The aim of the study was to analyze the insight of nine core dimensions of Polish PC system: “Economic conditions”, “Workforce”, “Accessibility”, “Comprehensiveness”, “Continuity”, “Coordination”, “Quality of care”, “Efficiency” and “Equity” and to identify the characteristics of the providing physicians that influence their perception of the quality of care.

**Methods:**

A cross-sectional study was conducted as part of an international QUALICOPC project. In Poland a nationally representative sample of 220 PC physicians was selected from the database of Polish National Health Fund by a stratified random sampling procedure. The research tool was a standardized 64-item questionnaire. Each of the respondents’ answers were assigned a numerical value ranging from−1 (extremely negative) to +1 (extremely positive). The quality indicators were calculated as an arithmetic mean of variables representing particular PC dimensions.

**Results:**

The mean scores for the majority of the dimensions had negative values. Accessibility of care was perceived as the best dimension, while the economic conditions were evaluated most negatively. Only a small part of variation in quality evaluation could be explained by physicians’ characteristics.

**Conclusions:**

The negative evaluation of primary care reflects the growing crisis in the health care system in Poland. There is an urgent need to apply complex recovery measures to improve the quality of primary care.

**Electronic supplementary material:**

The online version of this article (doi:10.1186/s12875-016-0550-8) contains supplementary material, which is available to authorized users.

## Background

Primary care (PC) is the first level of professional medical care, which allows patients to address their health needs. It deals with the majority of the population’s health problems [1]. A variety of studies have demonstrated that solid primary care systems are associated with effective health care delivery [2–7]. The WHO World Health Report 2008 emphasized the need for a renewal and strengthening of primary care [8]. Quality assurance in general practice is defined by the World Organization for National Colleges and Academies of General Practice/Family Medicine (WONCA) as “a continuous process of planned activities, based on performance review and setting explicit targets for good clinical practice with the aim of improving the actual quality of patient care” [9]. Avedis Donabedian defined the concept of healthcare quality as this three-level model: Structure-Process-Outcomes [10]. This model has been universally accepted and can also be used in the assessment of primary care [11–13]. Donabedian also paid attention to the importance of examining health care quality from more than one perspective [10]. According to Grol et al. there are three levels of quality analysis that need to be considered in primary care: the patients’ (customers’) perspective, views of different health care professionals (service providers) and administrative level (managers) [14].

Kringos et al. within the framework of Primary Health Care Activity Monitor for Europe (PHAMEU) project, performed in 2009/10, identified the following ten core dimensions of any primary care system: “Governance”, “Economic conditions”, “Workforce”, “Accessibility”, “Comprehensiveness”, “Continuity”, “Coordination”, “Quality of care”, “Efficiency” and “Equity”, allocating each of them either to the structure, process or outcome level, according to Donabedian’s categorization [15, 16]. The QUALICOPC (Quality and Costs of Primary Care in Europe) study coordinated by the Netherlands Institute for Health Services Research (NIVEL), following the same logic, aims to evaluate primary care systems in Europe at three levels of care: the system level of PC, provision level and the level of users of PC services. Data from the PHAMEU study were used as a part of the QUALICOPC project to collect evidence on PC at the system (structure) level. Surveys among general practitioners and their patients were carried out in 34 countries to gather mainly evidence at the process and outcome level. According to the QUALICOPC study protocol, the target response in GP survey was a nationally representative sample of 220 GPs per country, except for the four smallest countries (Cyprus, Iceland, Luxembourg and Malta), where the target was 75 [17]. As the dimension “Governance” was considered relatively distant from everyday reality in PC, it has not been included in the questionnaire for primary care physicians [18].

After the collapse of communism in Poland, like in many other Central and Eastern European countries, significant socioeconomic changes occurred. The Soviet-style centralized Semashko’s model of primary health care has been abandoned in order to build a more efficient system with the family physician serving a key role [19–21]. Although the new Polish model has been gradually built since the early nineties of the last century, it has not been comprehensively studied nor evaluated yet.

A large body of research in Poland addresses only single aspects of quality in primary care, mainly from the perspective of patients [22, 23]. In the light of the significance of multidirectional evaluation of quality in primary care, there is a need to gain insight in various viewpoints [10, 14]. Physicians as the main PC providers and the direct observers are able to provide trustworthy and reliable information about the functioning of primary care in Poland. Existing studies exploring the physicians’ perspective are limited in design; they mostly cover one health problem [24, 25]. In regards to Poland, there are a lack of publications that comprehensively asses the quality of PC from the general practitioners’ view.

The aim of this study was to evaluate the perception of primary healthcare quality in Poland by the physicians providing it and to assess the influence of doctors’ professional and demographic characteristics on their quality judgment.

## Methods

### Study design

A questionnaire based, cross-sectional study was performed as a part of an international project-Quality and Costs in Primary Care in Europe (QUALICOPC), and the detailed description of the methodology of the QUALICOPC study has already been published and is available elsewhere [17, 18]. The study was approved by the Jagiellonian University Bioethics Committee (approval number KBET/104/B/2011).

### Participants

Based on the QUALICOPC study protocol, the target response was a nationally representative sample of 220 general practitioners per country. As in Poland there are regional registers of primary care providers and regions differ in the size of the population and the number of practicing PC physician, we used a stratified, random sampling procedure. In the first stage, we selected 3 out of 16 Voivodeships (regions) of Poland. Next, taking into consideration the expected response rate of 50 %, 440 primary care practices out of 1454 in preselected regions were sampled from the registers of the National Health Fund-the exclusive health insurance company in Poland. In the third stage, one general practitioner was randomly selected from each practice and invited to take part in the study. From the sampling, we excluded primary care physicians who provided care to children exclusively, as the other part of the QUALICOPC project was a survey conducted among adult patients only. After the first sampling we did not manage to reach the target response, so we continued the sampling until the sample size of 220 PC physicians was obtained. Final participation rate was 33 %. We checked the representativeness of the participating physicians by comparing them with regard to age and gender to national statistics. This comparison showed that respondents were representative on age and gender for the population of primary care physicians in Poland. The questionnaires were delivered to and collected from study participants by trained fieldworkers.

### Data collection

The international questionnaire developed by QUALICOPC Consortium was used in the study. The questionnaire allowed the collection of data about three essential aspects of PC and the corresponding nine core dimensions. Thus “Structure” was characterized by “Economic conditions” and “Workforce development”, “Process” by “Accessibility”, “Comprehensiveness”, “Continuity” and “Coordination”, and “Outcome” by “Quality of care”, “Efficiency” and “Equity”. The questionnaire with an overview of the thematic content (assigned dimensions) of each of the questions has been already published elsewhere as an appendix available online [18]. We performed a cross-cultural adaptation of QUALICOPC questionnaire for use in Poland, which included five stages: (1) forward translation of the English version of the questionnaire by two independent translators, (2) comparison and analysis of the two translated versions by expert panel, (3) back translation, (4) instrument evaluation by the target population in a pilot study among 10 Polish primary care physicians and (5) psychometric testing. In the final Polish version, with the consent of project coordinator from the Netherlands Institute for Health Services Research, we have added three more questions about the background of the PC providers: the years of experience in PC, the specialization and the involvement in students’/residents’ training. In the last step of instrument adaptation we established psychometric properties of the Polish questionnaire. The internal consistency reliability was estimated by means of Cronbach’s alpha coefficient. The value of Cronbach’s alpha coefficients were respectively: for economic conditions 0.6; for workforce 0.61; for accessibility 0.63; for comprehensiveness 0.91; for continuity 0.79; for coordination 0.88; for quality of care 0.82; for efficiency 0.17; for equity 0.62. The construct validity we assessed through the analysis of internal structure of a test. Gamma coefficient was used to calculate the correlations between variables representing particular PC dimension and quality indicators. The conducted analysis showed significant correlations between variables and their assigned quality indicators. The correlations coefficients for particular variables in their assigned dimensions were respectively: in dimension “Economic conditions”: 0.14–0.91; in dimension “Workforce”: 0.54–0.64; in dimension “Accessibility”: 0.53–0.87; in dimension “Comprehensiveness”: 0.26–0.93; in dimension “Continuity”: 0.23–0.94; in dimension “Coordination”: 0.1–0.74; in dimension “Quality of care”: 0.67–0.99; in dimension “Efficiency”: 0.36–0.54; in dimension “Equity”: 0.32–0.88. We achieved good construct validity and acceptable reliability for each primary care dimension except “Efficiency”, in which Cronbach’s alpha fell below 0.6.

### Data analysis

To compare the quality of primary care dimensions, we developed quality indicators (QI) for all PC dimensions (except “Efficiency”). Every dimension was described by a set of nominal questions (variables) developed by the QUALICOPC consortium. The quality indicators were created on the basis of evaluation by an expert panel, consisting of experienced family physicians and researchers, who used an indirect structured consensus procedure-the Delphi method. A detailed description of the conducted consensus procedure is available in Additional file 1. All variables were rescaled to a scale ranging from−1 (extremely negative) to +1 (extremely positive). The quality indicators were calculated as an arithmetic mean (μ) of variables representing particular PC dimension. The used scale range [−1, 1] not only allowed the direct comparison between dimensions, but it also enabled to easily identify the primary care physicians’ opinions polarity.

In the analysis, we used two approaches to the quality indicators. Firstly, we analyzed them as interval variables ranging from−1 to +1. Secondly, from the interval variables we derived dichotomous variables: a “positive evaluation”, for the QI above 0 and a “negative evaluation”, for QI−1 to 0. In descriptive analyses, means, medians and ranges were calculated for interval variables and percentages for binary variables. To study the determinants of quality indicators, multiple linear regression and logistic regression were performed. In the regression models gender, place of work, composition of the practice population, experience in PC, specialization, involvement in students’/residents’ training, form of employment and other paid professional activities were considered as categorical explanatory variables. Age and patient list size were used as continuous covariates.

For statistical analysis Statistica 10 software package (Statsoft Inc.) was used. An alpha level of *p* = 0.05 was considered as tests of statistical significance.

## Results

### Characteristics of respondents

In total 220 PC physicians took part in the study. The detailed characteristics of the study’s participants in comparison with the national population of Polish PC physicians from the College of Family Physicians in Poland are presented in Table 1, where the majority of the respondents (64 %) were women and the mean age of the participants was 49,7 (SD = 8,7). Primary care practices, in which the respondents worked, were located with similar proportions in large cities, small and median size towns, and rural areas. The mean number of patients enlisted to the study’s participants was 2321 (SD = 988). 75 % of respondents had a specialization in family medicine, 18 % were internist and the rest had another type of medical specialization.

**Table 1:** Characteristics of respondents in comparison with the national population of Polish PC physicians from the College of Family Physicians in Poland

Feature	Respondents *n* = 220	National population
Gender		
Women n (%)	140 (64)	62 %
Men n (%)	80 (36)	38 %
Age in years		
mean (+/−SD)	49,7 (8,7)	46,4 (6,6)
[MIN; MAX]	[30; 82]	NA
Experience in PC		
0–15 years n (%)	120 (55)	mean (SD) 16,6 (7,3)
> 15 years n (%)	100 (45)	
Specialization		
Family Medicine (%)	59 (27)	NA
Family Medicine and other (%)	106 (48)	NA
Without Family Medicine (%)	55 (25)	NA
Place of work		
Big city n (%)	81 (37)	47 %
Small town n (%)	66 (30)	29 %
Village n (%)	73 (33)	23 %
Other physicians in the practice		
Yes n (%)	150 (68)	NA
No n (%)	70 (32)	NA
Patient list size		
mean (+/−SD)	2321 (988)	NA
[MIN; MAX]	[15; 5400]	NA
Elderly patients (>70 years of age) in the practice population		
Above avarege n (%)	62 (30)	NA
Average and below average n (%)	147 (70)	NA
Involvement in students’/residents’ training		
Yes n (%)	103 (47)	44 %
No n (%)	117 (53)	56 %
Form of employment		
Sole proprietorship (self-employment) n (%)	138 (63)	NA
Employment contract n (%)	82 (37)	NA
Other paid professional activities		
Yes n (%)	107 (49)	NA
No n (%)	113 (51)	NA

*NA* not available

### Quality indicators

**Fig. 1:**
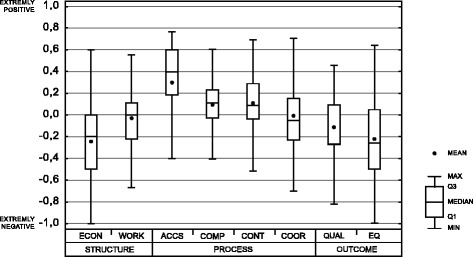
Perception of eight core dimensions of primary care. *ACCS-accessibility, COMP-comprehensiveness, CONT-continuity, COOR-coordination, ECON-economic conditions, EQ-equity, QUAL-quality, WORK-workforce, Q1 - the first quartile, Q3 - the third quartile, MIN - the minimum, MAX - the maximum*

In the studied primary care dimensions, the mean values of the quality indicator were as follows: economic conditions−0,24 (SD = 0,37); workforce−0,05 (SD = 0,29); accessibility 0,32 (SD = 0,32); comprehensiveness 0,09 (SD = 0,23); continuity 0,11 (SD = 0,28); coordination−0,02 (SD = 0,3); quality of care−0,12 (SD = 0,43); and equity−0,21 (SD = 0,35). Figure 1 presents distributions of quality indicators in particular dimensions of primary care.

**Fig. 2:**
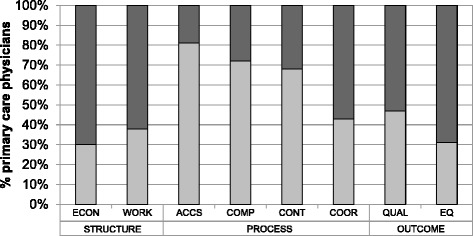
Positive and negative perceptions of eight core dimensions of primary care. *ACCS-accessibility, COMP-comprehensiveness, CONT-continuity, COOR-coordination, ECON-economic conditions, EQ-equity, QUAL-quality, WORK-workforce*  Negative evaluation: quality indicator <−1, 0>.  Positive evaluation: quality indicator (0, 1>.

The percentage of “positive evaluations” in particular dimensions ranged from 27 % for the dimension “Quality of care” to 81 % for “Accessibility”. Detailed data about positive and negative perceptions of each PC dimension are presented in Fig. 2.

### Factors associated with quality evaluation

**Fig. 3:**
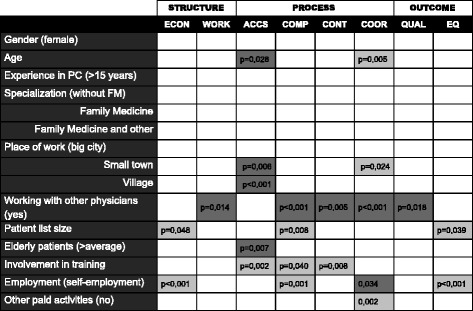
Linear regression model: associations of quality indicators in particular PC dimensions with physicians’ characteristics. *ACCS-accessibility, COMP-comprehensiveness, CONT-continuity, COOR-coordination, ECON-economic conditions, EQ-equity, QUAL-quality, WORK-workforce*  Negative association (*b* < 0; *p* ≤ 0,05).  Positive association (*b* > 0; *p* ≤ 0,05).  No association (*p* > 0;05).

The summary of linear regression models evaluating the associations between quality indicators and physicians’ characteristics is presented in Fig. 3.

The strongest identified relationships with the quality indicators in the studied primary care dimension were as follows: for economic conditions-the self-employment on contracts (*p* for Model = 0,001; *R2* = 0,161); for workforce-working alone in the practice without other physicians (*p* for Model = 0,003; *R2* = 0,073); for accessibility-place of work in a big city in comparison to work in more rural areas (*p* for Model < 0,001; *R2* = 0,331); for comprehensiveness-the self-employment (*p* for Model = 0,001; *R2* = 0,161); for continuity-involvement in training of students or residents (*p* for Model = 0,003; *R2* = 0,149); for coordination-lack of additional paid occupational activities besides work in primary care (*p* for Model < 0,001; *R2* = 0,238); for quality of care-working alone in the practice without other physicians (*p* for Model = 0,009; *R2* = 0,136); for equity the self-employment (p for Model < 0,001; *R2* = 0,17).

**Fig. 4:**
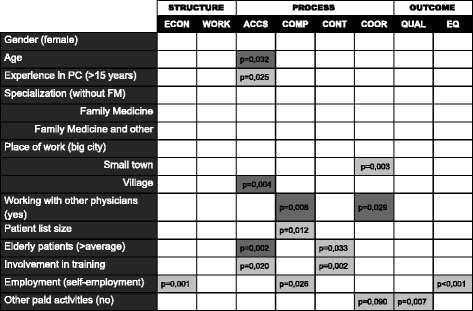
Logistic regression model: associations of positive quality evaluation in particular PC dimensions with physicians’ characteristics. *ACCS-accessibility, COMP-comprehensiveness, CONT-continuity, COOR-coordination, ECON-economic conditions, EQ-equity, QUAL-quality, WORK-workforce* Negative association (*OR* < 0; *p* ≤ 0,05).  Positive association (*OR* > 0; *p* ≤ 0,05).  No association (*p* > 0;05).

Figure 4 shows a summary of logistic regression models examining the associations of “positive evaluation” in particular PC dimensions with physicians’ characteristics.

The strongest determinants of positive quality evaluation in PC dimensions were: in economic conditions-the self-employed (*OR* = 4,30; 95%CI:1,81–10,2); in accessibility-involvement in training of students or residents (*OR* = 3,45; 95%CI:1,22–9,8); in comprehensiveness-the self-employed (*OR* = 2,57; 95%CI:1,12-5,89); in continuity-involvement in training of students or residents (*OR* = 3,35; 95%CI:1,55–7,23); in coordination-place of work in a small town in comparison to work in a big city (*OR* = 2,02; 95%CI:1,27–3,21); in quality of care-lack of additional paid occupational activities besides work in primary care *OR* = 2,57; 95%CI:1,3–5,1); in equity-the self-employment (*OR* = 4,15; 95%CI:1,86–9,25).

The detailed results of linear and logistic regression models for all dimensions are available in Additional file 2.

## Discussion

### Summary of main findings

Primary care physicians in Poland were generally very critical of the quality of primary care. The majority of primary care dimensions were evaluated negatively. The structure appeared to be the weakest aspect of the primary care quality, with economic conditions being the worst perceived among all primary care dimensions. Primary care process was identified as the strongest aspect of quality. Accessibility of care was the best perceived of all dimension. Polish primary care physicians reported positive experience with three out of four process describing dimensions (accessibility, comprehensiveness and continuity). Coordination scored the worst in the group. Pavlic et al. [26] analyzed the variability in process quality in family medicine among all countries participating in the QUALICOPC project. Coordination of care was identified as the weakest process quality indicators in family medicine. In outcome evaluation, quality of care scored better than equity, but both scores were negative.

Similar to Stokes et al. [27], we found that only a small part of variation in quality evaluation can be explained by physicians’ characteristics. In our study, self-employed physicians, those who had other paid professional activities besides their work in PC and who worked in practices where students and residents were trained, perceived the quality of particular PC dimensions in a more positive manner. Work in a group practice with other PC doctors or other medical specialists was associated with more negative evaluation of the PC quality.

### Strengths and limitations

This is the first study which presents a holistic assessment of the quality of primary care in Poland from the perspective of the physicians. The study was based on an international protocol and a uniform questionnaire, which allows direct comparisons between Polish primary care and PC systems in other countries. The international study design, however, has several limitations. First of all, despite random sampling, the results of the study cannot be generalized to Polish primary care physicians as a whole, because physicians who provide care solely to a pediatric population were excluded. Secondly, some of the topics in the questionnaire might not have been applicable for the Polish PC setting. Consequently, we validated the study tool and only one quality indicator (“Efficiency”) was non-reliable and was excluded from the final analyses. The construction of quality indicators itself was a potential source of bias, which we tried to eliminate using only validated questions and the Delphi method to achieve consensus about the importance and value of each of the indicators. It must also be taken into consideration that the present study was carried out in the first half of 2012, shortly after the time when Polish physicians were protesting against reforms in drug reimbursement regulations, which put doctors under new obligations and financial penalties. It cannot be excluded that this protest might have influenced physicians’ evaluations.

### Comparison with other studies

The final results of PHAMEU project based on analyses of available literature, governmental publications and experts’ consultations showed that primary care in Poland is characterized by good accessibility and coordination and a relatively weak structure (governance, economic conditions and workforce), which is in accordance with physician views from our study. Comprehensiveness in Polish primary care, evaluated positively by GPs in our study, is poorly developed according to the PHAMEU data. Overall, Poland was classified as a country with medium primary care strength [28].

The QUALICOPC project focused not only on physicians but also on their adult patients. It was found that in most of the countries, primary care shows one or more features with a medium or high level of patient-perceived improvement potential. In Poland, “Comprehensiveness” was indicated as a priority area with a medium level of patient-perceived improvement potential [29]. Nevertheless, in comparison to the doctors, Polish patients have explicitly more positive opinions about the quality of primary care. The PC dimensions best perceived among patients are: quality, equity and accessibility, while coordination and comprehensiveness get the worst but still positive evaluations [30]. Other Polish studies also showed high patient satisfaction with primary care [21]. Recent public opinion survey found that patients evaluated primary care most favorably among all health care services in Poland [31]. The discrepancy between positive evaluations of primary care by patients and negative assessment by doctors can be explained by patients’ bad experiences with secondary care, mainly due to limited access and poor interpersonal continuity of care (stable contact with chosen physician) [32]. The results of a systematic review of the literature by Sans-Corrales et al. confirmed that the attributes of family medicine such as accessibility, continuity of care, consultation time and the doctor-patient relationship are directly associated with patient satisfaction [33].

Physicians’ negative opinions about the quality of patient care can be observed worldwide. The five-country comparison conducted by Blendon et al. in Australia, Canada, New Zealand, the United Kingdom, and the United States revealed that physicians from all studied countries were concerned about a recent decline in quality of care. More than half of all physicians in the United States, Canada, and New Zealand, as well as 48 % of doctors in the United Kingdom and 38 % in Australia, expressed the feeling that their ability to deliver high-quality care has deteriorated over the past 5 years. According to two-thirds of Canadian physicians and around half of U.S. and New Zealand physicians, this problem will worsen in the future. In all five countries, doctors noticed the need for reforms to improve quality of care [34]. A need for changes in the health care systems was also found in a survey of primary care physicians in 11 countries conducted by the U.S. Commonwealth Fund. Only in the Netherlands and Norway most of general practitioners (60 % and 56 %, respectively) considered their health care system to be functioning well. Everywhere else, the majority of respondents agreed that fundamental changes are necessary in their health care systems [35].

### Interpretation of key findings

The introduction of family medicine in Poland in the early nineties of the last century was a demanding experience for Polish primary care physicians who had to adjust their professional life to the new model despite lack of examples in the former system [36]. A lack of support from the healthcare policy makers and a lack of unanimity among medical professionals in constructing a family medicine-based primary care system in compliance with European Union recommendations, have been observed for a few years and have caused a wane in the initial enthusiasm of transforming the Polish healthcare system [37]. Recent changes in Polish health care regulations, which allow specialists in internal medicine or pediatrics to work in the national health system as primary care physicians, are a step backwards in implementing a family medicine-based model [38]. In light of the current deterioration of the family medicine position in Poland, the negative opinions from primary care physicians are of no surprise.

Problems in primary care reflect a growing crisis of the health care system in Poland. In 2014, Poland had retained its 31st position at the bottom of the annual Euro Health Consumer Index (EHCI), scoring 10 points less than the previous year. According to EHCI, Poland is not able to keep an adequate level of healthcare despite a strong relative economic increase in comparison to other European countries [39]. A lack of political activism focusing on the strengthening of primary care might lead to further intensification of problems in the Polish healthcare system.

### Recommendations and future research proposal

In order to improve the quality of health care in Poland, there is a need to prepare and implement legal and organizational solutions, which would strengthen primary care in a actual and not just declarative way. The extension of Polish primary care physicians’ competencies is essential. However, shifting the tasks from hospital care and secondary care providers to primary care must be accompanied by an adequate increase in financial expenditure. Contracts for financing primary care health services from public funds should consider the economic conditions, in which primary care practices exist and not only specify the requirements to be fulfilled by the contract realization without taking into the account the effects for the PC provider. As international experience show, the most effective financing system of primary care services is the mixed system of payment with prevalence of capitation linked with fee for services and financial incentives for specific outcomes [40, 41]. Such system encourages physicians to increase desired activities, resulting in improving quality of health care [42].

In view of the growing crisis in the Polish health care system, the quality of care and methods of its improvement demand further studies. To gain a whole evaluation of primary care in Poland research among all primary care physicians, including physicians who provide care to children exclusively are needed. It also seems to be valuable to complement our results with qualitative studies using in-depth interviews or focus groups. These could allow Polish general practitioners to fully express their opinions about individual dimensions of provided care, which not always may be covered in quantitative research limited to particular survey questions.

In future it would be recommended to repeat the study with the presented research tool in order to directly monitor the changes in Polish primary care.

## Conclusions

In Poland, similarly to the trend observed worldwide, the quality of primary care is lowly evaluated by the physicians providing it. The features of physicians’ professional and demographic characteristics have hardly any influence on the perception of particular quality dimensions. The identification of main factors determining the physicians’ assessment of the quality of care requires further studies. The negative evaluation of primary care reflects the growing crisis in the health care system in Poland. There is an urgent need to apply complex recovery measures to improve the quality of primary care.

## Additional files

Additional file 1: Consensus procedure on quality indicators to assess the primary care in Poland. (DOCX 19 kb)
Additional file 2: Detailed results of linear and logistic regression models for all dimensions. (DOCX 55 kb)

## Abbreviations

ACCS: Accessibility
COMP: Comprehensiveness
CONT: Continuity
COOR: Coordination
ECON: Economic conditions
EQ: Equity
NIVEL: Netherlands Institute for Health Services Research
PC: Primary care
PHAMEU: Primary Health Care Activity Monitor for Europe
QI: Quality indicator
QUAL: Quality
QUALICOPC: Quality and Costs of Primary Care in Europe
WHO: World Health Organization
WONCA: World Organization for National Colleges and Academies of General Practice/Family Medicine
WORK: Workforce
