# Supplementary material for: Quality of primary health care in Poland from the perspective of the physicians providing it
Source: BMC Fam Pract. 2016 Nov 4;17:151. doi: 10.1186/s12875-016-0550-8 (PMC5096289; doi:10.1186/s12875-016-0550-8)
Supplement: Additional file 1: — Consensus procedure on quality indicators to assess the primary care in Poland. (DOCX 19 kb) [file 12875_2016_550_MOESM1_ESM.docx]

**Additional file 1: Consensus procedure on quality indicators to assess the primary care in Poland**

**Method**

A panel of professionals participated in a modified RAND Delphi procedure to compose a set of primary care quality indicators based on questionnaire for general practitioners from the QUALICOPC project. The consensus procedure included five consecutive steps: (1) Research team panel, (2) Selection of experts, (3) First round of Delphi, (4) Second round of Delphi, (5) Results analysis.

**Step 1: Research team panel**

The majority of questions from the QUALICOPC questionnaire for primary care physicians (apart from 7 questions about the providers’ background and the practice) described particular dimensions of primary care. The majority of questions were complex with many
sub-questions and included more than one variable. The research team from the Department of Family Medicine at the Jagiellonian University Medical College (JUMC) responsible for the Polish part of the QUALICOPC study conducted a review of literature and Polish legal regulations and prepared an initial proposition of rescaling all variables to a scale ranging from-1(extremely negative) to +1(extremely positive). A questionnaire for the first Delphi round was prepared. It included all variables from the QUALICOPC questionnaire with initially assigned numerical values.

**Step 2: Selection of experts**

A key selection criteria was that the expert had to have extensive knowledge about Polish primary care system. We invited 10 members of the College of Family Physicians in Poland, who cooperate with the Department of Family Medicine JUMC, to participate in the Delphi study. The participants provided written informed consent. All panelist were active general practitioners with at least 5 years of experience in primary care, which were involved in family medicine teaching of Polish medical students or residents. Four experts were researcher with experience in quality research in primary care.

**Step 3: First round of Delphi**

In the first Delphi-round, experts received a personal invitation for an online questionnaire. Panelists were asked to express their opinion about proposed rescaling of particular variables from the QUALICOPC questionnaire. They could agree or disagree with the research team proposal, or suggest other rescaling or exclusion of the variable from the further analysis giving a reason for exclusion. If at least 80% of experts agreed with the rescaling, the variable was considered to have face validity. Those variables, which were not rated valid in the first round of Delphi were included in the second round.

**Step 4: Second round of Delphi**

Based on the first Delphi-round results including experts suggestions, a new rescaling of the variables was proposed. A questionnaire for the second Delphi round was prepared. Panelists were asked to rescore agreement or disagreement in light of group's responses.

**Step 5: Results analysis**

The Delphi procedure allowed to prepare set of 182 variables on assumed interval scale out of 42 questions from the QUALICOPC questionnaire for general practitioners. The variables describing primary care were used to develop the quality indicators. The quality indicators were calculated as an arithmetic mean (µ) of variables representing particular PC dimension.

11 questions from the QUALICOPC questionnaire were excluded from the development of quality indicators, because the experts rate them as inappropriate in Polish health care system settings or were not able to rescale them to assumed scale. Two of the excluded questions ( about the form of employment and the other paid professional activities) were included in the final analysis as explanatory variables.

After data collection, in preliminary statistical analysis we calculated the quality indicators for all 9 studied dimensions (economic conditions, workforce, accessibility, comprehensiveness, continuity, coordination, quality of care, efficiency and equity), based on the variables established during Delphi study. However, checking psychometric properties, we achieved good construct validity and acceptable reliability for each primary care dimension except “Efficiency”, in which Cronbach’s alpha fell below 0.6. We excluded the dimension “Efficiency” from final analysis of quality indicators.
