# Supplementary material for: Quality of primary health care in Poland from the perspective of the physicians providing it
Source: BMC Fam Pract. 2016 Nov 4;17:151. doi: 10.1186/s12875-016-0550-8 (PMC5096289; doi:10.1186/s12875-016-0550-8)
Supplement: Additional file 2: — Detailed results of linear and logistic regression models for all dimensions. (DOCX 55 kb) [file 12875_2016_550_MOESM2_ESM.docx]

**Additional file 2: Detailed results of linear and logistic regression models for all dimensions**

**ECONOMIC CONDITIONS**

**Table 1** Linear regression model: association of “Economic conditions” quality indicator with physicians’ characteristics.

| Feature | Beta | b | p |
| --- | --- | --- | --- |
| Gender (female) | -0,028 | -0,022 | 0,68 |
| Age | -0,017 | -0,001 | 0,824 |
| Experience in PC (>15 years) | 0,001 | <0,001 | 0,997 |
| Specialization (without FM) |  |  |  |
| Family Medicine | -0,042 | -0,033 | 0,681 |
| Family Medicine and other | 0,004 | 0,003 | 0,969 |
| Place of work (big city) |  |  |  |
| Small town | -0,033 | -0,026 | 0,679 |
| Village | -0,001 | -0,001 | 0,991 |
| Working with other physicians (yes) | 0,045 | 0,035 | 0,538 |
| Patient list size | **0,142** | **<0,001** | **0,048** |
| Elderly patients (> average) | 0,103 | 0,083 | 0,143 |
| Involvement in training (yes) | -0,006 | -0,004 | 0,941 |
| Employment (self-employment) | **0,326** | **0,25** | **<0,001** |
| Other paid activities (no) | -0,04 | -0,03 | 0,559 |

**Table 2** Logistic regression model: association of positive quality evaluation in dimension “Economic condition” with physicians’ characteristics.

| Feature | OR (95%CI) | p |
| --- | --- | --- |
| Gender (female) | 0,99 (0,50-1,95) | 0,978 |
| Age | 0,99 (0,95-1,04) | 0,736 |
| Experience in PC (>15 years) | 0,86 (0,41-1,78) | 0,682 |
| Specialization (without FM) |  |  |
| Family Medicine | 0,69 (0,40-1,20) | 0,192 |
| Family Medicine and other | 0,90 (0,57-1,43) | 0,664 |
| Place of work (big city) |  |  |
| Small town | 0,98 (0,61-1,58) | 0,940 |
| Village | 0,80 (0,49-1,31) | 0,378 |
| Working with other physicians (yes) | 1,15 (0,55-2,42) | 0,702 |
| Patient list size | 1,00 (1,00-1,00) | 0,543 |
| Elderly patients (> average) | 1,75 (0,85-3,61) | 0,129 |
| Involvement in training (yes) | 0,85 (0,40-1,80) | 0,672 |
| Employment (self-employment) | **4,30 (1,81-10,20)** | **0,001** |
| Other paid activities (no) | 0,91 (0,47-1,76) | 0,79 |

**WORKFORCE**

**Table 3** Linear regression model: association of “Workforce” quality indicator with physicians’ characteristics.

| Feature | Beta | b | p |
| --- | --- | --- | --- |
| Gender (female) | 0,11 | 0,064 | 0,142 |
| Age | 0,01 | <0,001 | 0,906 |
| Experience in PC (>15 years) | -0,083 | -0,048 | 0,303 |
| Specialization (without FM) |  |  |  |
| Family Medicine | -0,139 | -0,087 | 0,194 |
| Family Medicine and other | -0,14 | -0,08 | 0,177 |
| Place of work (big city) |  |  |  |
| Small town | 0,007 | 0,005 | 0,93 |
| Village | -0,075 | -0,046 | 0,387 |
| Working with other physicians (yes) | **-0,191** | **-0,117** | **0,014** |
| Patient list size | -0,005 | <-0,001 | 0,946 |
| Elderly patients (> average) | -0,134 | -0,085 | 0,07 |
| Involvement in training (yes) | -0,55 | -0,032 | 0,512 |
| Employment (self-employment) | 0,065 | 0,039 | 0,42 |
| Other paid activities (no) | 0,03 | 0,017 | 0,681 |

**Table 4** Logistic regression model: association of positive quality evaluation in dimension “Workforce” with physicians’ characteristics.

| Feature | OR (95%CI) | p |
| --- | --- | --- |
| Gender (female) | 1,09 (0,58-2,04) | 0,784 |
| Age | 1,00 (0,96-1,04) | 0,919 |
| Experience in PC (>15 years) | 1,32 (0,67-2,57) | 0,423 |
| Specialization (without FM) |  |  |
| Family Medicine | 1,27 (0,77-2,08) | 0,346 |
| Family Medicine and other | 1,15 (0,75-1,76) | 0,511 |
| Place of work (big city) |  |  |
| Small town | 0,98 (0,62-1,53) | 0,918 |
| Village | 1,58 (0,99-2,53) | 0,057 |
| Working with other physicians (yes) | 1,80 (0,90-3,6) | 0,096 |
| Patient list size | 1,00 (1,00-1,00) | 0,481 |
| Elderly patients (> average) | 1,88 (0,93-3,81) | 0,078 |
| Involvement in training (yes) | 1,38 (0,68-2,81) | 0,376 |
| Forma zatrudnienia (Kontrakt) | 0,63 (0,30-1,29) | 0,204 |
| Inne płatne zajęcia (Nie) | 1,14 (0,62-2,09) | 0,664 |

**ACCESSIBILITY**

**Table 5** Linear regression model: association of “Accessibility” quality indicator with physicians’ characteristics.

| Feature | Beta | b | p |
| --- | --- | --- | --- |
| Gender (female) | 0,011 | 0,007 | 0,855 |
| Age | **-0,151** | **-0,005** | **0,028** |
| Experience in PC (>15 years) | 0,118 | 0,074 | 0,083 |
| Specialization (without FM) |  |  |  |
| Family Medicine | 0,057 | 0,039 | 0,527 |
| Family Medicine and other | 0,004 | 0,002 | 0,966 |
| Place of work (big city) |  |  |  |
| Small town | **-0,198** | **-0,135** | **0,006** |
| Village | **-0,381** | **-0,254** | **<0.001** |
| Working with other physicians (yes) | -0,003 | -0,002 | 0,958 |
| Patient list size | 0,012 | <0,001 | 0,849 |
| Elderly patients (> average) | **-0,171** | **-0,117** | **0,007** |
| Involvement in training (yes) | **0,225** | **0,14** | **0,002** |
| Employment (self-employment) | -0,101 | -0,066 | 0,142 |
| Other paid activities (no) | -0,034 | -0,021 | 0,582 |

**Table 6** Logistic regression model: association of positive quality evaluation in dimension “Accessibility” with physicians’ characteristics.

| Feature | OR (95%CI) | p |
| --- | --- | --- |
| Gender (female) | 1,16 (0,47-2,84) | 0,750 |
| Age | **0,94 (0,89-0,99)** | **0,032** |
| Experience in PC (>15 years) | **3,34 (1,16-9,57)** | **0,025** |
| Specialization (without FM) |  |  |
| Family Medicine | 1,15 (0,51-2,60) | 0,738 |
| Family Medicine and other | 1,05 (0,57-1,92) | 0,871 |
| Place of work (big city) |  |  |
| Small town | 0,66 (0,32-1,40) | 0,280 |
| Village | **0,36 (0,18-0,72)** | **0,004** |
| Working with other physicians (yes) | 1,23 (0,45-3,35) | 0,682 |
| Patient list size | 1,00(1,00-1,00) | 0,989 |
| Elderly patients (> average) | **0,23 (0,09-0,59)** | **0,002** |
| Involvement in training (yes) | **3,45 (1,22-9,80)** | **0,020** |
| Employment (self-employment) | 0,35 (0,11-1,05) | 0,061 |
| Other paid activities (no) | 0,82 (0,33-2,03) | 0,673 |

COMPREHENSIVENESS

**Table 7** Linear regression model: association of “Comprehensiveness” quality indicator with physicians’ characteristics.

| Feature | Beta | b | p |
| --- | --- | --- | --- |
| Gender (female) | -0,008 | -0,003 | 0,903 |
| Age | 0,115 | 0,003 | 0,100 |
| Experience in PC (>15 years) | 0,038 | 0,016 | 0,585 |
| Specialization (without FM) |  |  |  |
| Family Medicine | -0,029 | -0,014 | 0,756 |
| Family Medicine and other | 0,007 | 0,003 | 0,940 |
| Place of work (big city) |  |  |  |
| Small town | -0,051 | -0,024 | 0,475 |
| Village | 0,095 | 0,044 | 0,204 |
| Working with other physicians (yes) | **-0,283** | **-0,13** | **<0,001** |
| Patient list size | **0,174** | **<0,001** | **0,008** |
| Elderly patients (> average) | 0,086 | 0,041 | 0,179 |
| Involvement in training (yes) | **0,149** | **0,064** | **0,040** |
| Employment (self-employment) | **0,228** | **0,103** | **0,001** |
| Other paid activities (no) | 0,078 | 0,034 | 0,213 |

**Table 8** Logistic regression model: association of positive quality evaluation in dimension “Comprehensiveness” with physicians’ characteristics.

| Feature | OR (95%CI) | p |
| --- | --- | --- |
| Gender (female) | 1,15 (0,54-2,43) | 0,715 |
| Age | 0,99 (0,95-1,04) | 0,910 |
| Experience in PC (>15 years) | 1,83 (0,79-4,24) | 0,155 |
| Specialization (without FM) |  |  |
| Family Medicine | 0,65 (0,35-1,20) | 0,169 |
| Family Medicine and other | 1,03 (0,62-1,69) | 0,916 |
| Place of work (big city) |  |  |
| Small town | 0,59 (0,34-1,01) | 0,053 |
| Village | 1,33 (0,77-2,29) | 0,310 |
| Working with other physicians (yes) | **0,27 (0,10-0,71)** | **0,008** |
| Patient list size | **1,01(1,00-1,02)** | **0,012** |
| Elderly patients (> average) | 1,42 (0,60-3,35) | 0,425 |
| Involvement in training (yes) | 2,28 (0,99-5,27) | 0,053 |
| Employment (self-employment) | **2,57 (1,12-5,89)** | **0,026** |
| Other paid activities (no) | 1,08 (0,52-2,26) | 0,832 |

**CONTINUITY**

**Table 9** Linear regression model: association of “Continuity” quality indicator with physicians’ characteristics.

| Feature | Beta | b | p |
| --- | --- | --- | --- |
| Gender (female) | 0,064 | 0,038 | 0,359 |
| Age | 0,104 | 0,003 | 0,181 |
| Experience in PC (>15 years) | 0,080 | 0,046 | 0,296 |
| Specialization (without FM) |  |  |  |
| Family Medicine | -0,088 | -0,055 | 0,392 |
| Family Medicine and other | -0,158 | -0,090 | 0,112 |
| Place of work (big city) |  |  |  |
| Small town | 0,010 | 0,006 | 0,903 |
| Village | 0,042 | 0,026 | 0,610 |
| Working with other physicians (yes) | **-0,211** | **-0,128** | **0,005** |
| Patient list size | -0,035 | <-0,001 | 0,627 |
| Elderly patients (> average) | -0,005 | -0,003 | 0,946 |
| Involvement in training (yes) | **0,215** | **0,123** | **0,008** |
| Employment (self-employment) | -0,022 | -0,013 | 0,779 |
| Other paid activities (no) | 0,133 | 0,076 | 0,058 |

**Table 10** Logistic regression model: association of positive quality evaluation in dimension “Continuity” with physicians’ characteristics.

| Feature | OR (95%CI) | p |
| --- | --- | --- |
| Gender (female) | 1,05 (0,53-2,05) | 0,893 |
| Age | 0,99 (0,95-1,04) | 0,831 |
| Experience in PC (>15 years) | 0,80 (0,38-1,66) | 0,545 |
| Specialization (without FM) |  |  |
| Family Medicine | 0,77 (0,45-1,31) | 0,332 |
| Family Medicine and other | 0,79 (0,51-1,25) | 0,317 |
| Place of work (big city) |  |  |
| Small town | 1,10 (0,67-1,80) | 0,705 |
| Village | 1,05 (0,65-1,72) | 0,832 |
| Working with other physicians (yes) | 0,55 (0,25-1,19) | 0,131 |
| Patient list size | 1,00 (0,99-1,0) | 0,384 |
| Elderly patients (> average) | **2,36 (1,07-5,18)** | **0,033** |
| Involvement in training (yes) | **3,35 (1,55-7,23)** | **0,002** |
| Employment (self-employment) | 1,24 (0,58-2,66) | 0,573 |
| Other paid activities (no) | 1,32 (0,68-2,55) | 0,409 |

**COORDINATION**

**Table 11** Linear regression model: association of “Coordination” quality indicator with physicians’ characteristics.

| Feature | Beta | b | p |
| --- | --- | --- | --- |
| Gender (female) | 0,121 | 0,077 | 0,068 |
| Age | **0,207** | **0,007** | **0,005** |
| Experience in PC (>15 years) | 0,071 | 0,044 | 0,331 |
| Specialization (without FM) |  |  |  |
| Family Medicine | -0,057 | -0,038 | 0,557 |
| Family Medicine and other | -0,098 | -0,060 | 0,297 |
| Place of work (big city) |  |  |  |
| Small town | **0,172** | **0,116** | **0,024** |
| Village | 0,029 | 0,019 | 0,715 |
| Working with other physicians (yes) | **-0,273** | **-0,179** | **<0,001** |
| Patient list size | -0,048 | <-0,001 | 0,482 |
| Elderly patients (> average) | -0,008 | -0,006 | 0,899 |
| Involvement in training (yes) | 0,080 | 0,049 | 0,296 |
| Employment (self-employment) | **-0,157** | **-0,101** | **0,034** |
| Other paid activities (no) | **0,210** | **0,130** | **0,002** |

**Table 12** Logistic regression model: association of positive quality evaluation in dimension “Coordination” with physicians’ characteristics.

| Feature | OR (95%CI) | p |
| --- | --- | --- |
| Gender (female) | 1,66 (0,87-3,20) | 0,126 |
| Age | 1,01 (0,97-1,05) | 0,523 |
| Experience in PC (>15 years) | 1,48 (0,74-2,95) | 0,261 |
| Specialization (without FM) |  |  |
| Family Medicine | 0,60 (0,36-1,02) | 0,059 |
| Family Medicine and other | 1,09 (0,71-1,67) | 0,700 |
| Place of work (big city) |  |  |
| Small town | **2,02 (1,27-3,21)** | **0,003** |
| Village | 0,83 (0,52-1,32) | 0,432 |
| Working with other physicians (yes) | **0,45 (0,22-0,92)** | **0,029** |
| Patient list size | 1,00 (1,00-1,00) | 0,943 |
| Elderly patients (> average) | 1,17 (0,59-2,33) | 0,648 |
| Involvement in training (yes) | 1,24 (0,60-2,56) | 0,563 |
| Employment (self-employment) | 0,57 (0,27-1,19) | 0,133 |
| Other paid activities (no) | **1,72 (0,92-3,23)** | **0,090** |

**QUALITY OF CARE**

**Table 13** Linear regression model: association of “Quality of care” quality indicator with physicians’ characteristics.

| Feature | Beta | b | p |
| --- | --- | --- | --- |
| Gender (female) | 0,033 | 0,029 | 0,640 |
| Age | 0,134 | 0,007 | 0,087 |
| Experience in PC (>15 years) | 0,120 | 0,105 | 0,121 |
| Specialization (without FM) |  |  |  |
| Family Medicine | -0,019 | -0,018 | 0,854 |
| Family Medicine and other | -0,107 | -0,092 | 0,285 |
| Place of work (big city) |  |  |  |
| Small town | -0,105 | -0,099 | 0,194 |
| Village | 0,041 | 0,038 | 0,621 |
| Working with other physicians (yes) | **-0,178** | **-0,164** | **0,018** |
| Patient list size | -0,003 | <-0,001 | 0,963 |
| Elderly patients (> average) | -0,043 | -0,041 | 0,544 |
| Involvement in training (yes) | 0,092 | 0,080 | 0,257 |
| Employment (self-employment) | -0,092 | -0,083 | 0,241 |
| Other paid activities (no) | 0,138 | 0,120 | 0,051 |

**Table 14** Logistic regression model: association of positive quality evaluation in dimension “Quality of care” with physicians’ characteristics.

| Feature | OR (95%CI) | p |
| --- | --- | --- |
| Gender (female) | 1,17 (0,58-2,35) | 0,662 |
| Age | 1,03 (0,99-1,07) | 0,151 |
| Experience in PC (>15 years) | 1,39 (0,66-2,91) | 0,385 |
| Specialization (without FM) |  |  |
| Family Medicine | 0,81 (0,47-1,40) | 0,449 |
| Family Medicine and other | 0,73 (0,46-1,16) | 0,185 |
| Place of work (big city) |  |  |
| Small town | 0,71 (0,42-1,20) | 0,195 |
| Village | 1,14 (0,69-1,86) | 0,609 |
| Working with other physicians (yes) | 0,60 (0,28-1,29) | 0,192 |
| Patient list size | 1,00 (1,00-1,00) | 0,978 |
| Elderly patients (> average) | 0,52 (0,24-1,13) | 0,098 |
| Involvement in training (yes) | 0,62 (0,28-1,38) | 0,243 |
| Employment (self-employment) | 0,84 (0,39-1,80) | 0,654 |
| Other paid activities (no) | **2,57 (1,30-5,10)** | **0,007** |

**EQUITY**

**Table 15** Linear regression model: association of “Equity” quality indicator with physicians’ characteristics.

| Feature | Beta | b | p |
| --- | --- | --- | --- |
| Gender (female) | -0,031 | -0,022 | 0,653 |
| Age | 0,003 | <0,001 | 0,964 |
| Experience in PC (>15 years) | 0,01 | 0,007 | 0,900 |
| Specialization (without FM) |  |  |  |
| Family Medicine | -0,041 | -0,031 | 0,686 |
| Family Medicine and other | 0,027 | 0,019 | 0,784 |
| Place of work (big city) |  |  |  |
| Small town | -0,033 | -0,025 | 0,680 |
| Village | 0,010 | 0,008 | 0,901 |
| Working with other physicians (yes) | 0,034 | 0,025 | 0,644 |
| Patient list size | **0,148** | **<0,001** | **0,039** |
| Elderly patients (> average) | 0,095 | 0,073 | 0,174 |
| Involvement in training (yes) | 0,033 | 0,023 | 0,680 |
| Employment (self-employment) | **0,321** | **0,233** | **<0,001** |
| Other paid activities (no) | -0,028 | -0,019 | 0,686 |

**Table 16** Logistic regression model: association of positive quality evaluation in dimension “Equity” with physicians’ characteristics.

| Feature | OR (95%CI) | p |
| --- | --- | --- |
| Gender (female) | 0,94 (0,49-1,80) | 0,860 |
| Age | 1,00 (0,96-1,04) | 0,882 |
| Experience in PC (>15 years) | 0,74 (0,37-1,49) | 0,397 |
| Specialization (without FM) |  |  |
| Family Medicine | 0,73 (0,43-1,24) | 0,245 |
| Family Medicine and other | 1,07 (0,69-1,67) | 0,747 |
| Place of work (big city) |  |  |
| Small town | 0,86 (0,54-1,37) | 0,529 |
| Village | 0,91 (0,57-1,45) | 0,698 |
| Working with other physicians (yes) | 1,03 (0,51-2,07) | 0,943 |
| Patient list size | 1,00 (1,00-1,00) | 0,683 |
| Elderly patients (> average) | 1,48 (0,74-2,98) | 0,267 |
| Involvement in training (yes) | 0,86 (0,42-1,77) | 0,686 |
| Employment (self-employment) | **4,15 (1,86-9,25)** | **<0,001** |
| Other paid activities (no) | 0,95 (0,51-1,79) | 0,881 |
